# Spray-Drying Synthesis of Na_4_Fe_3_(PO4)_2_P_2_O_7_@CNT Cathode for Ultra-Stable and High-Rate Sodium-Ion Batteries

**DOI:** 10.3390/molecules30030753

**Published:** 2025-02-06

**Authors:** Jinri Huang, Ziheng Zhang, Daiqian Chen, Hesheng Yu, Yu Wu, Yuanfu Chen

**Affiliations:** State Key Laboratory of Electronic Thin Films and Integrated Devices, School of Integrated Circuit Science and Engineering, University of Electronic Science and Technology of China, Chengdu 611731, China; 202321310113@std.uestc.edu.cn (J.H.); 202111022820@std.uestc.edu.cn (Z.Z.); 202311311105@std.uestc.edu.cn (D.C.); 202411311215@std.uestc.edu.cn (H.Y.)

**Keywords:** spray-drying, Na_4_Fe_3_(PO_4_)_2_P_2_O_7_@CNT composite, long-term cyclic stability, sodium-ion battery

## Abstract

Iron-based phosphate is a promising cathode for sodium-ion batteries due to its low cost and abundant resources; however, the practical application is hindered by poor electronic conductivity, sluggish Na^+^ diffusion, and a lack of low-cost and scalable synthesis methods. To address such issues, herein, we present a low-cost and scalable spray-drying strategy to synthesize Na_4_Fe_3_(PO_4_)_2_P_2_O_7_@CNT (NFPP@CNT) hollow microspheres. The NFPP@CNT composite has the following advantages: highly conductive CNT can significantly improve the electronic conductivity of the cathode, and the flexible CNT-based microsphere architecture facilitates Na^+^ diffusion and guarantees excellent mechanical properties to mitigate structural degradation during cycling. These merits make the NFPP@CNT cathode display outstanding electrochemical performances: the NFPP@CNT-1% electrode demonstrates a high reversible capacity of 103.9 mAh g^−1^ at 0.1 C and maintains a very high capacity retention of 99.9% after 1000 cycles even at a high rate of 5 C.

## 1. Introduction

As population numbers swell, economic status enhances, and scientific innovation marches forward, the quest for energy is paramount among societal needs. Nowadays, lithium-ion batteries are famous for their long life along with energy density [1,2,3,4], and have become the darling of today’s society, as well as the most researched, mature, and widely used energy storage technology. However, there is a lack of resources for lithium on the earth, and the geographical distribution is uneven [5,6]. Consequently, the implementation of lithium-ion batteries in large-scale energy storage systems is deemed impractical. In the context of developing durable and cost-optimized electrochemical energy storage modalities, sodium-ion batteries (SIBs) are emerging as a compelling option to supplant lithium-ion technology, underpinned by the extensive reserves and reduced acquisition cost of sodium [7,8,9,10,11,12]. As alkali metal elements, sodium and lithium have similar atomic structures and chemical properties. In a battery system composed of Na/Na^+^ pairs and H/H^+^ pairs, the cell potential difference between the two electrodes is −2.71 V, which is close to the expected value of −3.04 V for a Li/Li^+^ and H/H^+^ couple configuration [13,14,15,16,17]. There are vast reserves of sodium in the crust of the earth, and most of it exists in seawater, so it is more convenient to extract, so its cost is lower, and sodium has obvious advantages over other alkali metal elements such as lithium and potassium for extensive energy storage solutions.

Across the spectrum of cathode materials that are suited for sodium-ion batteries, phosphate compounds containing iron have garnered significant interest because of their durable stability, eco-friendliness, and exceptional electrochemical efficiency [18,19]. Nevertheless, the low electronic conductivity and suboptimal cycle durability of iron phosphate-based materials constrain their utility in high-performance sodium-ion batteries [20,21]. To overcome these limitations, it is essential to engineer sophisticated electrode materials that boast improved stability and electrical conductivity.

Recently, a number of investigations have centered on optimizing the performance of iron-based phosphate electrodes by improving electrical conductivity and enhancing the structural strength of the electrodes. For example, Xia et al. [22] showed that CNT can effectively improve the conductivity and cycling stability of Na_3_Fe_2_(PO_4_)_3_ composite cathode. In addition, Xiong et al. [23] and Li et al. [24] explored the effects of surface modification and found that the appropriate doping of manganese or magnesium ions can markedly enhance the charge–discharge rate capability and the longevity of electrode materials. Wu et al. [25] discovered that replacing iron ions with appropriate manganese ions can considerably raise the operating voltage and cycle life of the battery. Through a strategy of structural tuning, Wu et al. [26] have found that the judicious doping of cadmium ions can effectively stabilize the crystal lattice and markedly enhance electronic conductivity. This optimization has led to the material demonstrating superior rate capability and an extended cycle life. However, despite the positive results of many studies, the high-rate performance and energy density of iron-based sodium-phosphate ion batteries still do not meet the standards for commercial application. In particular, there are still great challenges in the capacity retention rate after long charge–discharge cycles [27]. Furthermore, the reported synthesis methods (e.g., solid-state sintering [20] and the sol–gel method [28]) are difficult to realize in large-scale production, and it is difficult to control the composition/morphology/structure consistency and stability of NFPP.

To address the issues mentioned above, we present a low-cost and scalable spray-drying strategy to synthesize Na_4_Fe_3_(PO_4_)_2_P_2_O_7_@CNT (NFPP@CNT) hollow microspheres with many advantages. CNT can significantly improve the electronic conductivity of the cathode. The flexible CNT-based hollow microsphere architecture facilitates Na^+^ diffusion and guarantees structural stability during cycling. These merits make the NFPP@CNT cathode display outstanding electrochemical performances: the NFPP@CNT-1% electrode demonstrates a high reversible capacity of 103.9 mAh g^−1^ at 0.1 C and maintains very high capacity retention of 99.9% after 1000 cycles even at a high rate of 5 C.

## 2. Results and Discussion

**Figure 1:**
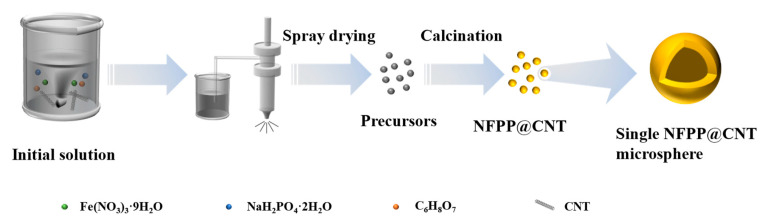
Illustration of the synthesis of NFPP@CNT.

Figure 1 illustrates the scalable production of NFPP@CNT composite with a unique hollow spherical shell structure, synthesized via a straightforward process combining spray-drying and one-step sintering.

The microstructural characteristics of the NFPP@CNT composite powder were elucidated utilizing SEM to ascertain the surface topography, while TEM was employed to gain insights into the internal morphology. Furthermore, HRTEM was utilized to scrutinize the integrity of the encapsulating layer and to resolve the atomic-scale lattice fringes within the composite.

**Figure 2:**
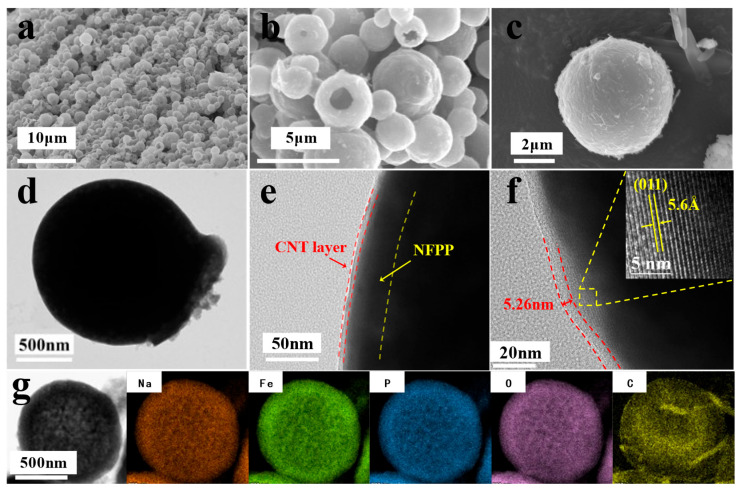
Morphological characterization of NFPP@CNT-1%: (**a**–**c**) SEM images; (**d**,**e**) TEM images; (**f**) HRTEM image; (**g**) typical SEM images and the corresponding elemental mappings of sodium (orange), iron (green), phosphorus (blue), oxygen (lilac), and carbon (yellow).

As shown in Figure 2a,b, the NFPP@CNT-1% electrode reveals a relatively uniform hollow spherical shell structure, with the diameter of the spherical shell ranging from 1 to 5 μm. It can be seen that the aggregated growth of NFPP@CNT-1% particles displays a smooth surface in the magnified SEM image of Figure 2c. Simultaneously, the image in Figure 2d clearly reveals the spherical shape of the particles under TEM observation. Figure 2e directly shows that it is a composite material composed of NFPP material and CNTs. Figure 2f depicts the thickness of the carbon layer is 5.26 nm. Moreover, the lattice fringes with a spacing of 5.6 Å shown in Figure 2f are in close alignment with the interplanar spacing of the (011) plane in the NFPP crystal structure, giving the evidence that the main component of the spherical shell is NFPP. Furthermore, the element mapping results were captured by EDS, as shown in Figure 2g. When combined with the TEM image of the same area, Na, Fe, P, and O are found to be uniformly and densely distributed across the sphere, indicating a more extensive distribution throughout the main body of the spherical shell. The distribution density of the C element is significantly lower than that of the previous four elements, suggesting its minimal encapsulation outside the NFPP core. The comprehensive findings from the conducted tests confirm that the composite material fabricated with success features NFPP spherical shells and CNT layers.

**Figure 3:**
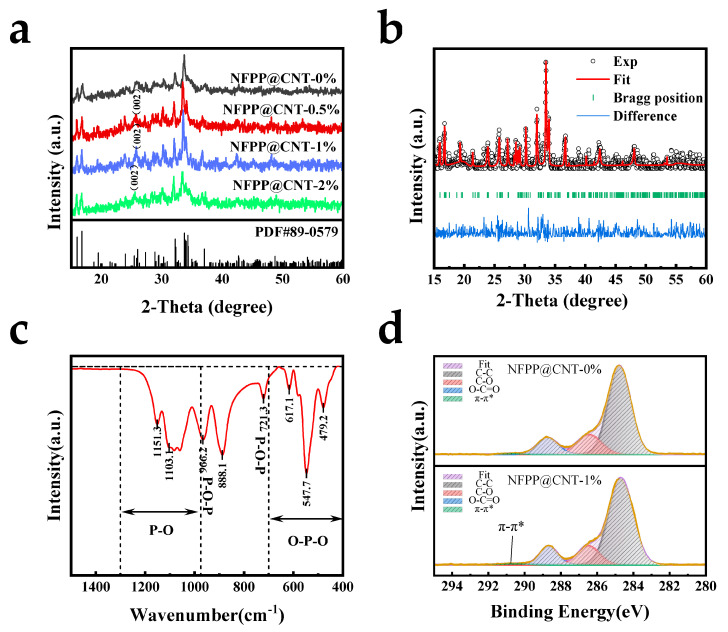
Composition analysis of NFPP@CNT: (**a**) XRD patterns of the NFPP@CNT with varied CNT contents from 0 to 2 wt%; (**b**) Rietveld refined XRD patterns of NFPP@CNT−1%; (**c**) the FTIR spectra of NFPP@CNT−1%; (**d**) the XPS spectra of C 1s of NFPP@CNT−1% and NFPP@CNT−0%.

Next, we performed XRD tests to conduct a preliminary investigation into the composition of the prepared NFPP@CNT samples. Figure 3a delineates that the crystallographic architectures of the NFPP@CNT materials are taxonomized within the orthorhombic system, corresponding to the space group designation Pn2_1_a. Additionally, the X-ray diffraction pattern of the specimen aligns with the reference data from the standard Powder Diffraction File (PDF) card. And, alternative diffraction peaks were not evident in the observation, confirming that CNT can not affect the original structure of the NFPP. Moreover, an increase in the CNT content leads to a subtle alteration in the NFPP lattice structure, with the diffraction peaks migrating to the direction of lesser angles, indicating that the NFPP lattice experiences slight expansion as the CNT content increases. This may be due to the bonding of CNTs with the matrix, which leads to lattice expansion. The Rietveld refinement was employed to study the specific impact of the CNT on the lattice parameters, with the results shown in Figure 3b. The refinement results indicate that the lattice parameters are a = 18.10354 Å, b = 6.53515 Å, and c = 10.57885 Å. From the XRD patterns of the samples, it can be observed that the 0.5%, 1%, and 2% samples all show the characteristic graphite peak of CNT near 26°, confirming the successful incorporation of CNTs, while the 0% sample does not exhibit a distinct peak. The infrared spectrum depicted in Figure 3c reveals two distinct peaks at approximately 721.3 and 966.2 cm^−1^, which correspond to the symmetric and antisymmetric P-O-P stretching vibrations of the P_2_O_7_ group, respectively, while the O-P-O bending and P-O stretching vibrations in the PO_4_ group are correspond to the multiple peaks ranging from 400 to 700 cm^−1^ and 975 to 1300 cm^−1^ [29]. From the XPS spectra (Figure S1), peaks corresponding to the elements Na, Fe, P, O, and C can be observed in the samples. Figure 3d shows the XPS profiles of the C 1s energy level for the NFPP@CNT-1% and NFPP@CNT-0% electrodes. As shown in the figure, both of the profiles can be divided into several weak peaks together with a stronger one. Aside from the primary peak located at 284.8 eV, which is indicative of C-C bonding, there are observable auxiliary peaks at 286.5 and 288.7 eV. These secondary peaks indicate the incorporation of oxygenated functional entities within the material, particularly the C-O and O=C-O bonds [20,29]. Moreover, in the NFPP@CNT-1% electrode, a very weak peak was observed at 290.5 eV, which is consistent with the π-π^*^ bond on the graphite ring [30], indicating that CNTs have been successfully introduced into the sample.

**Figure 4:**
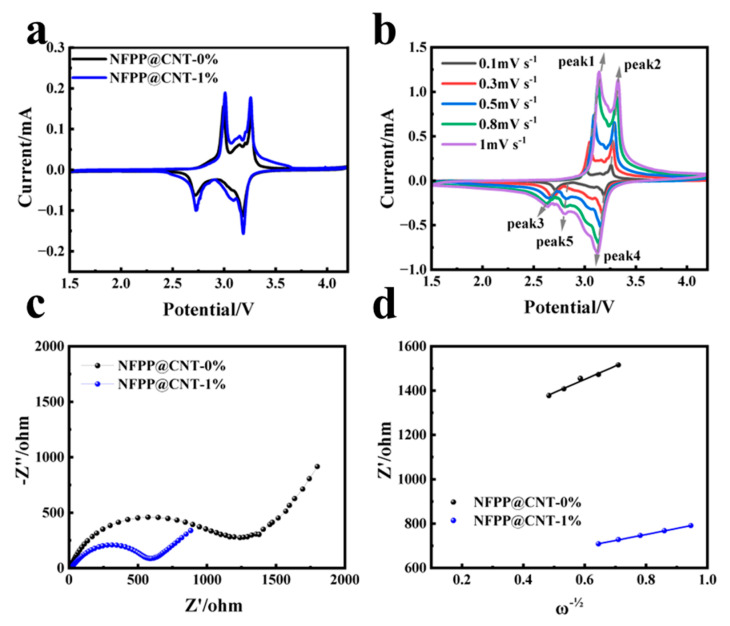
Electrochemical performance test of NFPP@CNT: (**a**) CV curves for NFPP@CNT−0% and 1% electrodes; (**b**) CV curves of NFPP@CNT−1% electrode at different scan rates (0.1, 0.3, 0.5, 0.8, and 1 mV s^−1^); (**c**) the EIS profiles after 100 cycles; (**d**) the relationship between Z′ and ω^−1/2^ in the low-frequency region.

To study the electrochemical properties, we conducted cyclic voltammetry (CV) measurements at a scan rate of 0.1 mV s^−1^, along with constant current charging and discharging cycles that were performed across a potential range of 1.5 to 4.2 V with respect to the Na/Na^+^ electrode. As shown in Figure 4a and Figure S2, the 0% and 1% electrodes exhibit distinct redox peaks at 3.0/2.7 V and 3.3/3.2 V, which correspond to the insertion and deinsertion of Na^+^ at different sites [31]. The presence of multiple minor oxidation peaks between 3.0 and 3.3 V may be related to the rearrangement of Na^+^ sites [32]. In contrast, the reduction peaks of the 0.5% and 2% electrodes at 2.7 V shift to 2.6 V, indicating poorer reversibility of the electrochemical reaction. This results in a larger potential difference at 3.0/2.7 V compared to the 0% and 1% samples. Notably, the redox peaks of the NFPP@CNT−1% electrode are sharper, which implies enhanced electrochemical reversibility for the NFPP@CNT−1% electrode. Furthermore, the NFPP@CNT−1% electrode prominently showcases an elevated and more pronounced peak current magnitude, indicative of its superior electrochemical activity, indicating that CNT can enhance the intensity of the redox reactions, which is manifested as an increase in the cycling specific capacity. We tested the CV curves of the NFPP@CNT−1% sample at different scan rates (Figure 4b) and calculated the pseudocapacitive behavior of the electrode. The calculation results are shown in Figure S3. The results indicate that the reaction kinetics of the NFPP@CNT−1% electrode material are more inclined towards pseudocapacitive properties. Subsequently, we tested the EIS spectra of both samples after 100 cycles, the results are shown in Figure 4c. The Zview software was employed to fit the Nyquist plots of the samples. The interfacial transfer resistance of the NFPP@CNT−0% sample is 1277 Ω, which is much higher than that of the NFPP@CNT−1% sample at 616.1 Ω, suggesting that the enhanced interfacial electron transfer capabilities of the electrode surface, attributed to the introduction of highly conductive CNT. Additionally, the ion diffusion coefficients within the battery were calculated based on the Warburg region of the Nyquist plot, along with the calculation method. The relationship between Z’ and ω^−1/2^ in the Warburg region is shown in Figure 4d, and the calculation results are presented in Table S1 [26,33]. The data reveals that the NFPP@CNT−1% sample possesses a superior Na^+^ diffusion coefficient as opposed to the NFPP@CNT−0% sample, suggesting that CNTs help to boost the sodium ion diffusion kinetics. The results of the tests have substantiated that the composite, characterized by its unique CNT-clad NFPP spherical shell architecture, significantly enhances the surface electron conductivity of the electrode and markedly accelerates the diffusion kinetics of sodium ions.

**Figure 5:**
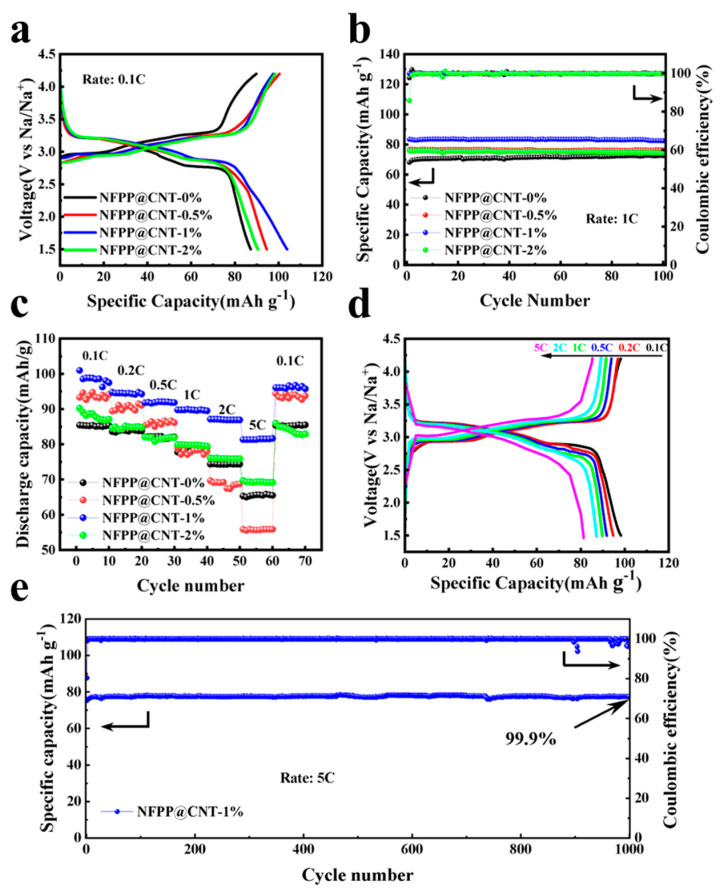
Electrochemical performance test of NFPP@CNT: (**a**) Galvanostatic charge and discharge profiles at a current rate of 0.1 C (1 C = 120 mA g^−1^); (**b**) the cycling performances and corresponding coulombic efficiencies at 1 C; (**c**) rate performance of NFPP@CNT with varied CNT contents; (**d**) the corresponding charge and discharge curves of NFPP@CNT−1% at various rates; (**e**) the cycling performances and corresponding coulombic efficiencies of NFPP@CNT−1% at 5 C.

Using sodium metal as the anode and the prepared NFPP@CNT electrode as the cathode, we conducted continuous current charge–discharge cycles on the half-cell within a voltage range of 1.5 to 4.2 V relative to the Na/Na^+^ redox couple to study the electrochemical performance of the NFPP@CNT material. The data are depicted in Figure 5a–e Among them, Figure 5a shows the electrochemical cycling performance of the battery at a current rate of 0.1 C. From the figure, it can be observed that the discharge specific capacities of the batteries with the CNT contents of 0%, 0.5%, and 2% are 87.3, 94.6, and 90.6 mAh g^−1^, respectively. At a CNT content of 1% by weight, the specific discharge capacity attains a value of 103.9 mAh g^−1^, indicating that the modification effect is optimal when the CNT weight fraction is 1%. Additionally, distinct voltage plateaus during the charge and discharge processes are seen at 3.3/3.2 V and 3.0/2.7 V, respectively, which are in alignment with the two major redox events detected in the CV curve. The charge–discharge profiles in Figure 5b document the results of 100 cycles at a 1 C rate. It is evident that the NFPP@CNT−1% electrode exhibits superior cyclic stability, with a stable specific capacity reaching approximately 85 mAh g^−1^. In contrast, the NFPP@CNT−0% electrode has the least specific capacity, stabilizing at about 70 mAh g^−1^. During the entire cycling process, all the samples maintain a high coulombic efficiency (coulombic efficiency is defined as the ratio of the amount of charge released during discharge to the amount of charge input during the previous charging cycle, usually expressed as a percentage) of over 95%. Additionally, after 100 cycles, all the samples demonstrate an impressive capacity retention of more than 98%. As illustrated, the capacity retention rate of all four samples is above 98% after 100 cycles. Figure 5c,d illustrate the performance of the four electrodes as a function of rate, as well as the associated charge–discharge profiles at various current rates. The NFPP@CNT−1% electrode exhibits very high rate performance, with reversible capacities of 98.22, 94.77, 91.79, 89.78, 87.12, and 81.32 mAh g^−1^ at different rates of 0.1, 0.2, 0.5, 1, 2, and 5 C, respectively. Notably, as shown in Figure 5c, at the exceptionally high rate of 5 C, the NFPP@CNT−1% electrode exhibits a pronounced advantage compared to the other samples, with measured specific capacities of 66, 56, and 69 mAh g^−1^ for the respective control tests. All the test results indicate that the performance improvement effect of 1% CNT is the most significant. This may be because the 2% content of CNTs is prone to agglomeration in the matrix, which leads to a decrease in the bonding with the matrix. In contrast, the 0.5% content of CNTs, due to the relatively low addition amount, is insufficient in forming a conductive network and enhancing the matrix properties. Therefore, compared to 0.5% and 2%, 1% is the most suitable ratio for CNT to be uniformly dispersed on the surface of NFPP spheres and to form the uniform layer of cladding, and the corresponding NFPP@CNT−1% exhibits the optimal performance. Moreover, following 1000 cycles at a high charge–discharge rate of 5 C, the NFPP@CNT−1% electrode maintains a reversible specific capacity retention of 99.9% as shown in Figure 5e, indicating that the NFPP@CNT−1% material has high cycling stability at the high rate of 5 C (the summary and comparison of NFPP material performance reported in the literature are shown in Table S2 [25,31,32,34,35,36]). The aforementioned test outcomes confirm that the composite, which integrates NFPP with a CNT layer, enhances the electrode’s electrical conductivity at the surface level and greatly facilitates the movement of sodium ions.

The introduction of CNTs may also influence the composition of the cathode-electrolyte interphase (CEI), promoting the formation of a thinner and more uniform CEI layer and reducing the accumulation of organic decomposition products [37]. CNTs are known for their electrical conductivity and mechanical stability. These properties enable them to form conductive networks on the surface of the cathode material, reducing charge transfer impedance and thereby minimizing interfacial polarization. Lower impedance helps maintain the integrity of the CEI, reducing capacity fade caused by interfacial instability [38]. Additionally, the incorporation of CNTs enhances structural stability, which is conducive to the formation of a more uniform and stable CEI layer. This stability reduces the decomposition of the electrolyte and side reactions [39].

## 3. Materials and Methods

### 3.1. Material Synthesis

In this work, Na_4_Fe_3_(PO_4_)_2_P_2_O_7_@CNT composites with different CNT contents were prepared by spray-drying. The carbon nanotubes, which are commercially available multi-walled carbon nanotubes (MWCNTs), were ultrasonically dispersed in 80mL deionized water for 1 h (the carbon nanotube content was 0%, 0.5%, 1%, and 2% of the theoretical product weight), and Fe(NO_3_)_3_·9H_2_O (15 mmol, 6.06 g), NH_4_H_2_PO_4_ (20 mmol, 2.3 g), Na_2_C_2_O_4_ (10 mmol, 1.34 g), and anhydrous citric acid (10 mmol, 1.9212 g) were dissolved in the above CNT dispersion and stirred for 2 h. The precursor powder was obtained through spray-drying, and the inlet/outlet temperatures of spray-drying were 180 °C and 80 °C, respectively. The obtained precursor material was heated to 500 °C at 5 °C min^−1^ in an Ar atmosphere, kept for 10 h, and naturally cooled to room temperature to obtain Na_4_Fe_3_(PO_4_) _2_P_2_O_7_@ multi-walled carbon nanotube material (denoted as NFPP@CNT).

### 3.2. Characterizations

The synthesized NFPP@CNT samples were ground into powders and characterized by powder XRD (CuKα 30 kV 25 mA) with a scanning speed of 2° min^−1^ at the range of 15–60°. The morphology of the NFPP@CNT hybrid material was elucidated utilizing the scanning electron microscopy (SEM) and conventional transmission electron microscopy (TEM) methodologies. Subsequently, the lattice images were characterized by high-resolution transmission electron microscopy (HRTEM) techniques. The chemical identity of the synthesized compound as Na_4_Fe_3_(PO_4_)_2_P_2_O_7_ was ascertained by Fourier transform infrared spectroscopy (FTIR), while the embedding of CNTs within the compound was authenticated by X-ray photoelectron spectroscopy (XPS) examinations.

### 3.3. Electrochemical Performances

The electrochemical characteristics of the NFPP@CNT composite electrodes were evaluated utilizing CR2025 coin cells. The cathodic material was fabricated through a coating process. The synthesized Na_4_Fe_3_(PO_4_)_2_P_2_O_7_@CNT composite was blended with a conductive additive (acetylene black) and a binder (polyvinylidene fluoride, PVDF) in a proportion of 7:2:1 by weight. The PVDF was a 40 mg ml^−1^ PVDF solution dispersed in N-Methylpyrrolidone (NMP), which was prepared in advance. The mixture was stirred for 6 h to form a homogeneous slurry. Then, the slurry was coated on carbon-coated aluminum foil, and dried in a vacuum oven at 110 °C for 6 h. There was about 1–1.5 mg cm^−2^ of the active material weight on the pole piece. The anode of the battery was a sodium metal sheet. The electrolyte consisted of 1 M NaClO_4_ dissolved in ethylene carbonate (EC) and diethyl carbonate (DEC) solutions at a 1:1 volume ratio with the addition of 5 wt% fluoroethylene carbonate (FEC). In order to assemble the batteries, the procedure was carried out within an argon-purged glove box environment, and the assembled batteries were left to rest for 6 h and then subjected to electrochemical data testing. The electrochemical charge and discharge characteristics of the NFPP@CNT composite electrodes were examined over a potential range of 1.5 to 4.2 V (vs. Na/Na^+^) at a controlled room temperature of 25 °C. For the purpose of measuring the cyclic voltammetry (CV) and electrochemical impedance spectroscopy (EIS) spectrum, we use the electrochemical workstation (CHI660D), with the voltage of the CV test in a range of 1.5–4.2 V, while the frequency of the EIS test ranging from 100 kHz to 0.1 Hz after 100 charge–discharge cycles.

## 4. Conclusions

In summary, we have synthesized NFPP@CNT-1% spherical shells through scalable spray-drying combined with a one-step sintering method. The introduction of CNTs has markedly increased the electronic transport properties at the electrode surface, yielding outstanding results in facilitating the swift diffusion of sodium ions during the charge and discharge processes. The synthesized NFPP@CNT-1% electrode displays a striking 103.9 mAh g^−1^ of reversible specific capacity when subjected to a 0.1 C rate cycle. Moreover, the electrode maintains a reversible specific capacity exceeding 80 mAh g^−1^ when the rate is raised to 5 C, and it possesses an exceptional cycle stability, retaining 99.9% of its initial capacity after 1000 cycles at 5 C. The simplicity of the spray-drying technique, along with the budget-friendly aspect of the original materials, coupled with the excellent safety and performance characteristics of the NFPP@CNT composite material, make it suitable to meet the demands of advanced sodium-ion batteries that are seeking cost-effective large-scale energy storage solutions.

## Data Availability

The raw data supporting the conclusions of this article will be made available by the authors on request.

## Supplementary Materials

The following supporting information can be downloaded at: https://www.mdpi.com/article/10.3390/molecules30030753/s1, Figure S1: XPS survey spectrum of the NFPP@CNT-1% material; Figure S2: CV plots of (a) NFPP@CNT-0.5% and (b) 2% electrodes; Figure S3: CV plots of (a) NFPP@CNT-0.5% and (b) 2% electrodes; Table S1: The EIS fitting data and calculated **D*_Na+_*** for different samples; Table S2: Summary and Comparison of NFPP Material Performance Reported in the Literature.
