# Supplementary material for: Spray-Drying Synthesis of Na4Fe3(PO4)2P2O7@CNT Cathode for Ultra-Stable and High-Rate Sodium-Ion Batteries
Source: Molecules. 2025 Feb 6;30(3):753. doi: 10.3390/molecules30030753 (PMC11819995; doi:10.3390/molecules30030753)
Supplement: Supplementary file 1 [file molecules-30-00753-s001.zip › molecules-3426436-supplementary.pdf]

Spray-drying synthesis of $Na_4Fe_3(PO4)_2P_2O_7$@CNT cathode for ultra-stable and high-rate sodium-ion batteries

Jinri Huang, Ziheng Zhang, Daiqian Chen, Hesheng Yu, Yu Wu*, and Yuanfu Chen*

School of Integrated Circuit Science and Engineering, and State Key Laboratory of Electronic Thin Films and Integrated Devices, University of Electronic Science and Technology of China, Chengdu 610054, China

*Corresponding authors.

E-mails: 619142937@qq.com(Y Wu); yfchen@uestc.edu.cn (Y Chen)

Postal address: School of Integrated Circuit Science and Engineering, and State Key Laboratory of Electronic Thin Films and Integrated Devices, University of Electronic Science and Technology of China, Chengdu 610054, China.

The test of diffusion coefficient of sodium ions

In the low-frequency region of the Nyquist plot, we can calculate the diffusion coefficient of sodium ions within the battery. The value can be determined using the following equation[1, 2]:

$$\mathrm{D}N_a^+ = \frac{R^2T^2}{2A^2n^2F^4C^2\sigma^2}$$

Where C can be calculated through the density and molar mass of the composite material (i.e., C = material density / molar mass), F is the Faraday constant (96500 C mol^{-1}), A is the electrode surface area, T is the absolute temperature (298.15 K), R is the gas constant (8.314 J K^{-1} mol^{-1}), n is the number of electrons per molecule in the redox process, and σ is the Warburg factor related to Z' (the real part of the battery impedance, in ohms) and ω (frequency, in Hz). The calculation formula for the Warburg factor σ is:

$$Z' = Re + R_{ct} + \sigma\omega^{-\frac{1}{2}}$$

In the above equation, R_e represents the electrolyte resistance, R_{ct} is the charge transfer resistance, and ω is the angular frequency in the low-frequency region. That is, σ can be obtained through linear fitting in the low-frequency region. The specific method is as follows: Draw a line with a slope of 45° on the graph and make it tangent to the Warburg impedance. Select five points in the tangential section to create a $\omega^{-1/2}$ - Z' plot, where ω = 2πf. Perform a linear fit on the $\omega^{-1/2}$ - Z' plot, and the slope obtained from this linear fit is σ.

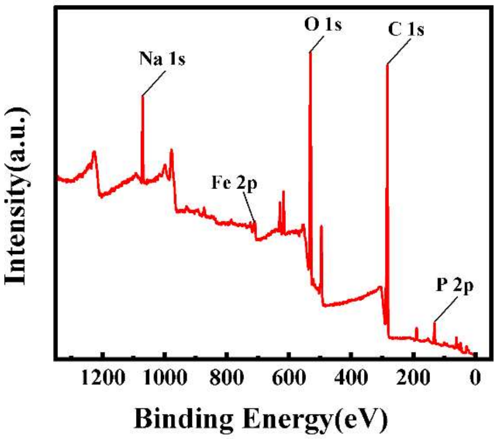

Fig. S1 XPS survey spectrum of the NFPP@CNT-1% material

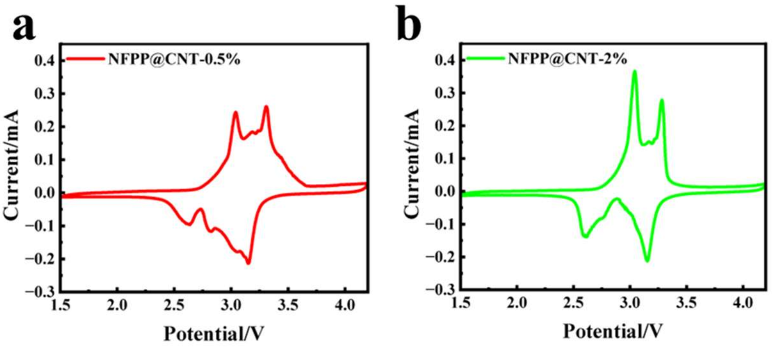

Fig. S2 CV plots of (a) NFPP@CNT-0.5% and (b) 2% electrodes

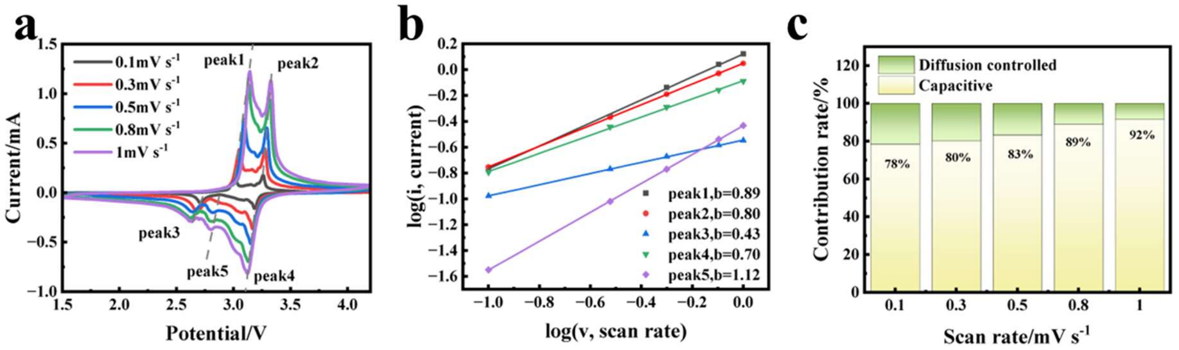

Fig. S3 Pseudo-capacitance calculation results (a) CV profiles of NFPP@CNT-1% electrode at different scanning rates (0.1, 0.3, 0.5, 0.8 and 1.0 mV s^{-1}); (b) Linear fitting results of log *I* versus log *v* plots; (c) The contribution ratio of capacitive capacities in NFPP@CNT-1%.

Table S1. The EIS fitting data and calculated $\mathbf{D}_{Na+}$ for different samples

**Sample**	**R_s/Ω**	**R_{ct}/Ω**	**σ/ (Ω s^{-1})**	**D_{Na+}/ (×10^{-13})**
NFPP@CNT-0%	6.69	1277.00	601.58	0.44
NFPP@CNT-1%	14.17	616.10	271.35	5.59

Table S2. Summary and Comparison of NFPP Material Performance Reported in the Literature

Material	Rate	Number of Cycles	Capacity Retention	Reference
NFPP/HC	10C	4000	63.5%	3
HE-NFPP	0.5A g^{-1}	1000	91.6%	4
NFPPCHG	10C	500	65.9%	5
NFPP@rGO	2C	500	82.1%	6
NFPP-HE	10C	1500	82.3%	7
$NFM_{1.2}PP$	5C	1000	79.1%	8
Our work	5C	1000	99.9%	This work

References

1. Wu, F.; Ma, H.; Ye, X.; Wu, S. Structural modulation of $Na_4Fe_3(PO_4)_2P_2O_7$ via cation engineering towards high-rate and long-cycling sodium-ion batteries. *Journal of Colloid and Interface Science* **2025**, *679*, 132-140. DOI: 10.1016/j.jcis.2024.09.206.

2. Chen, T.-R.; Wu, Z.-G.; Xiang, W.; Wang, E.-H.; Wu, C.-J.; Chen, M.-Z.; Guo, X.-D.; Zhong, B.-H. The influences of sodium sources on the structure evolution and electrochemical performances of layered-tunnel hybrid $Na_{0.6}MnO_2$ cathode. *Ceramics International* **2017**, *43* (8), 6303-6311. DOI: 10.1016/j.ceramint.2017.02.036.

3. Pu, X.; Wang, H.; Yuan, T.; Cao, S.; Liu, S.; Xuc, L.; Yang, H.; Ai, X.; Chen, Z.; Cao, Y. $Na_4Fe_3(PO_4)_2P_2O_7$/C nanospheres as low-cost, high-performance cathode material for sodium-ion batteries. *Energy Storage Materials* **2019**, *22*, 330-336. DOI: 10.1016/j.ensm.2019.02.017.

4. Jiang, N.; Wang, X.; Zhou, H.; Wang, Y.; Sun, S.; Yang, C.; Liu, Y. Achieving Fast and Stable Sodium Storage in $Na_4Fe_3(PO_4)_2(P_2O_7)$ via Entropy Engineering. *Small* **2024**, *20* (26). DOI: 10.1002/smll.202308681.

5. Li, X.; Meng, Y.; Xiao, D. Three-Dimensional Holey Graphene Modified $Na_4Fe_3(PO_4)_2(P_2O_7)$/C as a High-Performance Cathode for Rechargeable Sodium-Ion Batteries. *Chemistry-a European Journal* **2023**, *29* (12). DOI: 10.1002/chem.202203381.

6. Shi, K.; Yang, W.; Wu, Q.; Yang, X.; Zhao, R.; She, Z.; Xie, Q.; Ruan, Y. Boosting the fast electrochemical kinetics of $Na_4Fe_3(PO_4)_2(P_2O_7)$ via a 3D graphene network as a cathode material for potassium-ion batteries. *New Journal of Chemistry* **2023**, *47* (21), 10153-10161. DOI: 10.1039/d3nj00553d.

7. Ge, X.; Li, H.; Li, J.; Guan, C.; Wang, X.; He, L.; Li, S.; Lai, Y.; Zhang, Z. High-Entropy Doping Boosts Ion/Electronic Transport of $Na_4Fe_3(PO_4)_2(P_2O_7)$/C Cathode for Superior Performance Sodium-Ion Batteries. *Small* **2023**, *19* (37). DOI: 10.1002/smll.202302609.

8. Wu, H.; Wen, T.; Chen, L.; Ding, Y.; Pu, X.; Cao, Y.; Chen, Z. Understanding the Role of Mn Substitution for Boosting High-Voltage $Na_4Fe_{3-x}Mn_x(PO_4)_2P_2O_7$ Cathode in Sodium-Ion Batteries. *Small Methods* **2024**, ; Early Access. DOI: 10.1002/smtd.202400642.
